# Improvement of Mechanical Properties of Plasma Sprayed Al_2_O_3_–ZrO_2_–SiO_2_ Amorphous Coatings by Surface Crystallization

**DOI:** 10.3390/ma12193232

**Published:** 2019-10-02

**Authors:** Jan Medricky, Frantisek Lukac, Stefan Csaki, Sarka Houdkova, Maria Barbosa, Tomas Tesar, Jan Cizek, Radek Musalek, Ondrej Kovarik, Tomas Chraska

**Affiliations:** 1 Institute of Plasma Physics, The Czech Academy of Sciences, Za Slovankou 1782/3, Prague 182 00, The Czech Republic; lukac@ipp.cas.cz (F.L.); csaki@ipp.cas.cz (S.C.); tesar@ipp.cas.cz (T.T.); cizek@ipp.cas.cz (J.C.); musalek@ipp.cas.cz (R.M.); chraskat@ipp.cas.cz (T.C.); 2 Department of Materials, Faculty of Nuclear Sciences and Physical Engineering, Czech Technical University in Prague, Prague 115 19, The Czech Republic; ondrej.kovarik@fjfi.cvut.cz; 3 Research and Testing Institute Pilzen, Pilsen 301 00, The Czech Republic; houdkova@vzuplzen.cz; 4 Fraunhofer IWS, 01277 Dresden, Germany; maria.barbosa@iws.fraunhofer.de

**Keywords:** amorphous, nanocrystalline, wear resistant, Vickers microhardness, plasma spraying

## Abstract

Ceramic Al_2_O_3_−ZrO_2_−SiO_2_ coatings with near eutectic composition were plasma sprayed using hybrid water stabilized plasma torch (WSP-H). The as-sprayed coatings possessed fully amorphous microstructure which can be transformed to nanocrystalline by further heat treatment. The amorphous/crystalline content ratio and the crystallite sizes can be controlled by a specific choice of heat treatment conditions, subsequently leading to significant changes in the microstructure and mechanical properties of the coatings, such as hardness or wear resistance. In this study, two advanced methods of surface heat treatment were realized by plasma jet or by high energy laser heating. As opposed to the traditional furnace treatments, inducing homogeneous changes throughout the material, both approaches lead to a formation of gradient microstructure within the coatings; from dominantly amorphous at the substrate–coating interface vicinity to fully nanocrystalline near its surface. The processes can also be applied for large-scale applications and do not induce detrimental changes to the underlying substrate materials. The respective mechanical response was evaluated by measuring coating hardness profile and wear resistance. For some of the heat treatment conditions, an increase in the coating microhardness by factor up to 1.8 was observed, as well as improvement of wear resistance behaviour up to 6.5 times. The phase composition changes were analysed by X-ray diffraction and the microstructure was investigated by scanning electron microscopy.

## 1. Introduction

Thermally sprayed ceramic coatings are widely used in industry to provide mechanical, chemical and thermal protection. Coatings are prepared by introducing the feedstock material, most often in a form of powder, into a hot plasma jet, where it is melted and propelled towards a prepared substrate. After their impact at the substrate, the molten particles flatten and solidify in a form of disk-like platelets called splats. Plasma spraying inherently possesses extremely high cooling rates of the particles, in the range 10^3^–10^6^ K/s, thereby frequently giving rise to a formation of non-equilibrium phases, microstructures of fine columnar grains [1] or even amorphous phases [2].

Due to its low price as well as good chemical and wear resistance, Al_2_O_3_ is often used as a material of the first choice for protecting metallic parts from wear and corrosion. Additionally, Al_2_O_3_ properties can be further significantly improved when it is mixed with other components. For example, Al_2_O_3_−Y_2_O_3_ and Al_2_O_3_−TiO_2_ composite coatings possess higher wear resistance than pure Al_2_O_3_ [3,4], while the addition of ZrO_2_ increases the coatings’ toughness [5].

Another way to improve the coating properties is a preparation of nanocrystalline or sub-micron microstructure: nanocrystalline materials are characterized by a microstructural length or grain size of up to about 100 nm, while microstructure having grain sizes from ∼0.1 to 0.3 μm are classified as submicron materials [6]. It was shown that nanocrystalline materials possess better mechanical properties than their coarse-grained counterparts [7]. For instance, a nanocrystalline Inconel 718 coating deposited by high velocity oxygen fuel (HVOF) method exhibited a significant increase in hardness (by approximately 60%) over that of the Inconel 718 control sample [8]. Another results published in [9] showed that decreasing the grain size of Al_2_O_3_ feedstock powder from ∼50 μm down to 300 nm increased the tensile adhesion strength of the deposited coating by a factor of three and the coating wear resistance was increased by a factor of ten. Nanostructured zirconia coatings deposited by plasma spraying in the study by Chen et al. [10] showed that the wear rates of the nanostructured coatings were about 40% of those of traditional zirconia coatings under loads from 20 to 80 N. Owing to these outstanding performances, targeted applications have been successfully implemented for hard and wear-resistant ceramic coatings in industrial sectors in the past decade [6].

Suspension plasma spraying has been intensively studied as a reliable method of preparation of coatings with such fine, nanometric-grain microstructure. In the early suspension experiments, nano-sized particles were used. However, their tendency towards agglomeration as well as associated difficult handling and potential health risk of using such suspensions [11] triggered a shift to using sub-micrometric particles instead. Even though the coatings prepared by suspension plasma spraying route can often surpass the coatings prepared from dry powders [9], their industrial application remain rather scarce at the moment, owing to difficulties in the coating preparation and relatively low deposition rate (coating thickness increase per torch pass) compared to dry powder plasma spraying [12,13].

An alternative approach to preparation of nanocrystalline coatings is based on deposition of amorphous coatings from coarse powders and their subsequent heat treatment in order to induce growth of nanocrystalline grains in the microstructure. Deposition of such amorphous microstructures can be relatively easily implemented through the rapid solidification of the particles in plasma spraying, provided the cooling rates are sufficiently high to fully suppress crystalline growth. It has been previously reported that materials with near-eutectic composition can solidify as fully or partially amorphous solids, and this finding was widely used for preparation of e.g. metallic glasses [14] or amorphous ceramics, such as Al_2_O_3_−Y_2_O_3_, Al_2_O_3_–ZrO_2_ and Al_2_O_3_–ZrO_2_–SiO_2_ systems, as presented in [3,15,16,17,18,19]. Transformation of amorphous coating of the ternary Al_2_O_3_–ZrO_2_–SiO_2_ system into nanocrystalline coating was successfully reported in our previous studies [19,20], where an improvement of the mechanical properties was also described. In both studies, amorphous atmospheric plasma sprayed materials Al_2_O_3_–ZrO_2_ and Al_2_O_3_–ZrO_2_–SiO_2_ were isothermally heat treated at various temperatures above the crystallization temperature (980 °C), forming nanocrystallites embedded in the amorphous matrix. Size of the nanocrystallites was strongly dependent on the heat treatment temperature and resulted in different mechanical properties, such as hardness and flexural strength [20].

The traditional way of heat treatment of amorphous materials would be furnace treatment; however, it is not applicable for coatings on metallic substrates because of substrate oxidation, grain growth and even possible spallation of the coating, caused by the differences in the substrate–coating coefficients of thermal expansion. In this paper, we present two alternative methods of heat treatment of amorphous Al_2_O_3_−ZrO_2_−SiO_2_ coatings deposited by atmospheric hybrid water stabilized plasma torch (WSP-H). The study is focused on laser and plasma surface heat treatment techniques, i.e., industrially relevant methods with high potential. As opposed to furnace annealing (used as a reference method for free standing ceramic parts), both of these methods enable on-site modification of the coating properties without inducing any detrimental changes to the metallic substrate material and enable surface heat treatment of large-sized components, such as paper-mill rollers. Upon the treatment, the phase composition and microstructure changes were evaluated by XRD and SEM, respectively, and the associated mechanical response of the coating to heat treatment was studied by measuring the Vickers microhardness and Pin on Disc wear resistance tests.

## 2. Materials and Methods

Feedstock Al_2_O_3_−ZrO_2_−SiO_2_ ceramic powder was obtained by crushing commercially available bulk material Eucor (Eutit Ltd., Stara Voda, Czech Republic). The powder was sieved into a sprayable size distribution with *D*_50_ = 89 μm, as measured by particle size analyser Mastersizer 3000 (Malvern, UK). Using the EDX analysis, the near eutectic composition of the feedstock powder was determined as 49% Al_2_O_3_, 31% ZrO_2_ and 19% SiO_2_ (all ± 2%). The hybrid water stabilized plasma torch WSP-H 500 (ProjectSoft, Hradec Kralove, Czech Republic) was used for plasma spraying onto S235 steel substrates with dimensions 50 × 30 × 10 mm^3^. The torch was operated at 500 A (∼150 kW) and 15 slpm argon flow rate. In addition to argon, the torch consumes about 20 g/min of demineralized water, which is evaporated and ionized to supply the plasma with hydrogen and oxygen ions (for detailed information about spraying with the WSP-H torch, refer to [21,22]). The stand-off distance was set to 350 mm and powder was injected radially into plasma jet at a feeding distance (i.e., the distance of the powder injection point from the torch exit nozzle) of 35 mm and powder feed rate of 10 kg/h (167 g/min).

Prior to the deposition, the substrates were grit blasted by alumina grit (*Ra* = 8.1 ± 0.3 μm) and mounted to a revolving carousel. The substrates temperature during the deposition was measured by infra-red camera TIM160 (Micro-Epsilon, Ortenburg, Germany) facing the substrates’ front side, as well as by a K-type thermocouple inserted into a hole drilled from the back-side of one of the samples and reaching 1 mm under the coated surface. The substrates were preheated by 3 cycles of plasma torch with deactivated powder feeding. To prepare a coating with a 1.5 mm thickness, 11 successive plasma torch cycles were needed. One deposition cycle consisted of three up and down strokes and was followed by an extensive cooling. Each deposition cycle was manually triggered when temperature measured by the thermocouple dropped to 250 °C.

**Figure 1:**
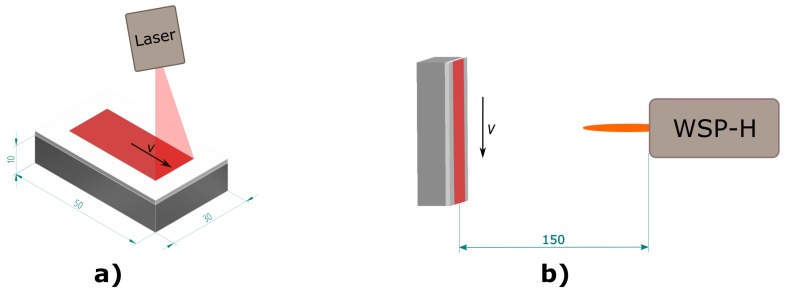
Schematic illustration of samples surface heat-treatment by laser (**a**) and WSP-H plasma torch (**b**).

To facilitate an accurate determination of the heat treatment variants’ influence, the as-sprayed coatings were polished down using a P600 diamond disc. Subsequently, the samples were subjected to one of two methods of surface heat-treatment: laser or plasma. The laser treatment was performed using a high power diode laser (Laserline GmbH, Muhlheim-Karlich, Germany) with maximal output power of 9 kW, λ = 915–1030 nm, 1000 μm fibre diameter, 400 mm focal length and laser focus diameter 7.5 mm. The laser transverse velocity and power were varied to obtain 15 different laser heat-treatment conditions in total. On the other hand, high enthalpy plasma generated heat produced by the plasma torch offers a quick and readily available alternative since the heat treatment can be performed directly after the coating deposition using the same plasma torch that was used for the coating deposition. The torch transverse velocity and power were modified, in order to prepare 6 different plasma heat-treatment conditions. The samples were mounted into a stationary sample holder and heat-treated by a single pass of the plasma torch at a stand-off distance of 150 mm. A schematic illustration of the two surface heat-treatment methods is provided in Figure 1.

Stripped-off ceramic coatings were then also prepared from the as-sprayed samples by grinding off the substrates. These coatings were used for measurement of the thermal expansion using vertical dilatometer Setsys 16/18 (Setaram, Caluire-et-Cuire, France) and to determine the crystallization onset temperatures using a Bahr STA 504 differential thermal analyser (Bahr, Hullhorst, Germany). In addition to the two surface heat-treatment methods, complimentary furnace annealing of the stripped-off ceramic samples was carried out. The furnace Entech EEF 5/16-HV (Entech, Angelholm, Sweden) was first preheated to the temperature 1050 °C and the samples were then inserted for a specified time. Such samples, isothermally treated in the whole volume (as opposed to gradient heating during laser or plasma treatment), were used as a reference set.

Metallographic samples of all specimens were prepared using Tegramin-25 automated polishing system (Struers, Willich, Denmark). The polished cross-sections were observed using a scanning electron microscope EVO MA 15 (Carl Zeiss SMT, Oberkochen, Germany) equipped with XFlash 5010 energy-dispersive spectrometer EDX (Bruker, Hamburg, Germany). Porosity of the coating was evaluated from seven SEM micrographs with nominal magnification 500× using semi-automatic thresholding procedure in ImageJ software (National Institutes of Health, Bethesda, MD, USA). Vickers microhardness profiles were measured on the polished sample cross-sections throughout the coating thickness using Q10A+ universal hardness tester (Qness, Golling an der Salzach, Austria), using the load of 300 g and dwell time 10 s. The average value of Vickers microhardness was calculated from at least 5 indents. Phase composition was evaluated on the free surfaces of the samples by powder X-ray diffractometer (XRD) D8 Discover (Bruker, Hamburg, Germany), using Cu anode and equipped with 1D detector. The degree of crystallinity, size of coherently diffracting domains (CDD) and microstrains were evaluated by quantitative Rietveld analysis of the acquired XRD spectra. Broadening of the diffraction peaks and background fitting were analysed using TOPAS V5 software (Bruker AXS, Hamburg, Germany). It was assumed that the effects of small crystallite size and microstrains contribute to broadening of Lorentzian and Gaussian components of pseudo-Voigt function, respectively [23].

Tribological properties were measured by CSEM High Temperature Tribometer (Anton Paar GmbH, Graz, Austria) by dry sliding Pin on Disc test according to ASTM G99 05 standard. The tests were carried out at room temperature, in air atmosphere (31% relative humidity) without lubrication using alumina counterpart ball (6 mm diameter) with 10 N normal load, 0.1 m·s^−1^ speed and measured distance of 110 m in 5000 cycles (track radius 3.5 mm). The wear tracks profiles were measured by profilometer P-6 Profiler (KLA-Tencor, Milpitas, CA, USA), at four different places, and the wear volume was calculated.

## 3. Results

### 3.1. As-Sprayed Samples

**Figure 2:**
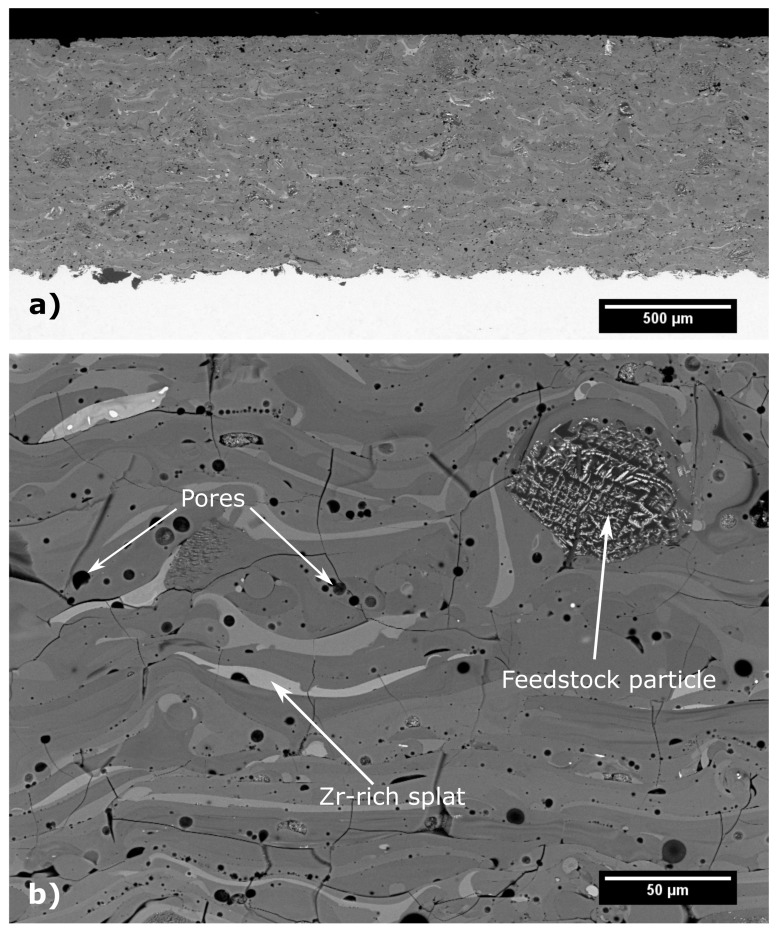
Microstructure of as-sprayed Al_2_O_3_−ZrO_2_−SiO_2_ coating cross-section: (**a**) coating overview; (**b**) magnified view.

**Figure 3:**
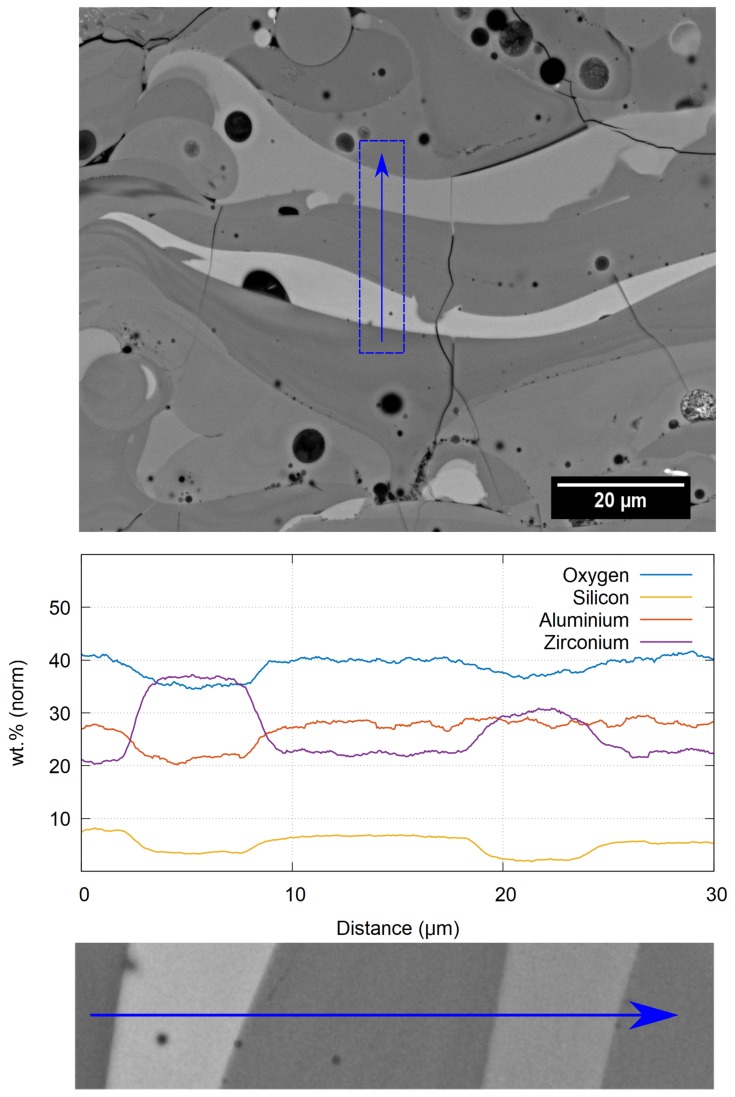
Local EDX line analysis of the splats in the as-sprayed material.

Cross-sections, prepared from the as-sprayed coating, were analysed using SEM in Back Scattered Electron (BSE) mode to observe the microstructure and overall coating quality. As seen from Figure 2a, the coatings evenly covered the substrate and adhered well to it with neither delaminations nor vertical cracks observed. The average chemical composition of the coating in wt.%, as evaluated by EDX, was: 51 ± 1 Al_2_O_3_, 33 ± 2 ZrO_2_, 13 ± 1 SiO_2_, with traces of Fe- and Na-oxides. From the magnified view in Figure 2b, it can be observed that individual splats differed significantly in the shade of grey, which is caused by variations of their chemical composition. The brighter the splat, the more ZrO_2_ it contains, while darker splats are richer in Al_2_O_3_, as was confirmed by EDX analysis (see Figure 3). Apart from the compositional variations of the splats, some unmelted feedstock particles were observed embedded in the as-sprayed coating. These particles can be easily recognized by their original eutectic microstructure (Figure 2b), which was retained from the feedstock powder. The amount of unmelted particles within the coating was 4.4 ± 0.9%, as evaluated by the image analysis. The magnified view also shows short micro cracks within the coating, with an average length of about 80 μm. The total porosity of the as-sprayed coating, as evaluated by the image analysis, was 4.7 ± 0.2% and consisted mainly of globular pores with average size around 8 μm^2^.

The XRD measurement of the samples free surfaces showed that the as-sprayed coatings were mainly amorphous, with only about 8% of crystalline phases present, assumedly dominantly formed by unmelted particles as described above.

### 3.2. Thermal Properties

**Figure 4:**
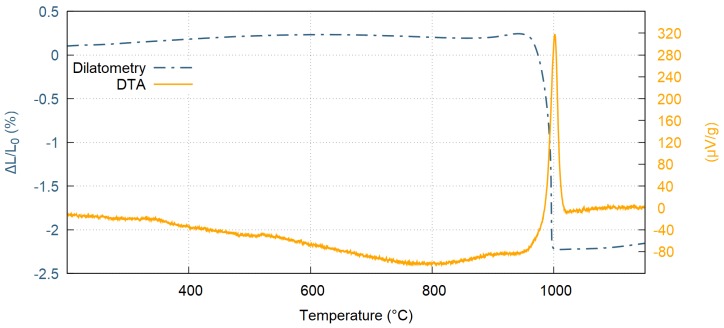
Measurement of differential thermal analysis (DTA) and thermal dilatometry of free standing Al_2_O_3_−ZrO_2_−SiO_2_ coatings.

Free standing ceramic samples were prepared from the as-sprayed coatings by grinding off the substrate. These samples were used for Differential Thermal Analysis (DTA) to obtain crystallization temperature of the amorphous samples. The DTA showed onset crystallization peak at 984 °C, and the crystallization was fully finished at 1015 °C, as shown in Figure 4. Moreover, thermal dilatometry was measured suggesting a rapid linear shrinkage of 2.47% observed at crystallization temperature (blue dash-and-dot line in Figure 4). The average value of the coefficient of thermal expansion (CTE) before crystallization, determined from measurement of displacement, was (3.5 ± 0.7) × 10^−6^ K^−1^ and changed to (6.1 ± 0.4) × 10^−6^ K^−1^ after crystallization. When the identical sample was subjected to a second measurement of displacement, CTE remained constant within the full temperature range up to 1300 °C, suggesting that the primary crystallization is an irreversible transformation.

### 3.3. Heat Treatment

The as-sprayed samples were surface heat-treated by laser or plasma. Additionally, two free standing ceramic samples were furnace heat-treated at 1050 °C (i.e., slightly above the determined crystallization temperature) with dwell time 1 and 5 min, for further comparison with the surface heat-treated samples. Parameters of all heat treatment conditions are listed in Table 1. Notation of the samples in Table 1 is as follows: AS—as sprayed sample, F#—Furnace, L#—Laser and P#—Plasma heat treated samples. Please consider that plasma torch used in the experiment had power of 100–150 kW. Therefore, to prevent melting of the samples, the transverse velocities used for plasma torch treatment had to be significantly higher than the ones used for laser treatment.

**Figure 5:**
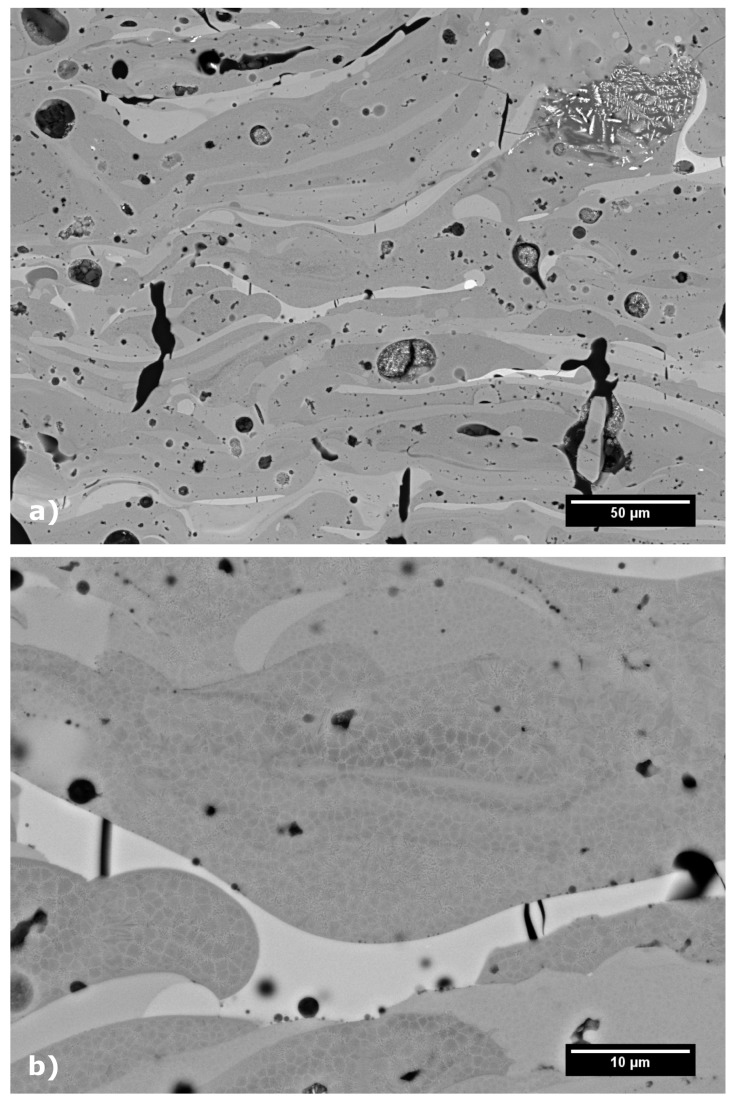
Cross-section of furnace heat-treated sample at 1050 °C and 5 min dwell (**a**); magnified view (**b**).

The heat treatment of the samples led to significant changes in the microstructure, as well as the phase composition. Furnace treatment of the samples resulted in shrinkage of about 1.6%, as measured by a vernier caliper on the samples before and after heat treatment. Moreover, heat treatment led to closing of internal microcracks, and merging of small globular voids into larger pores, as shown in Figure 5a. A more detailed study in Figure 5b showed a formation of polygonal crystallites within individual splats (cf. the amorphous microstructure in Figure 2b). In most splats, the crystallites consisted of δ-Al_2_O_3_ (dark grains), surrounded by t-ZrO_2_ (observed by SEM and confirmed by the analysis of XRD patterns). Solid state crystallization took place, with various kinetics, depending on the chemical composition of each splat, resulting in formation of δ-Al_2_O_3_ grains with size between 0.4–2 μm.

**Figure 6:**
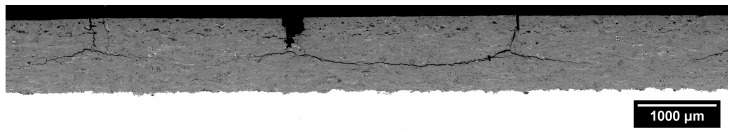
Cross-section of plasma surface heat treated sample P2 containing a major vertical and horizontal cracks, classified as type 3 in this paper. Such cracking yields the procedure unusable for applications.

Surface heat treatment by laser and plasma also resulted in the coating crystallization. The degree of crystallization and microstructural changes in the sample depended on treatment parameters. For some samples, both laser and plasma treatment resulted in cracking of the coating. Based on this, the samples were categorized into four groups, depending on the morphology of newly developed cracks as follows: 0—no new cracks present, 1—short (<200 μm) vertical cracks, 2—long vertical cracks and 3—long vertical cracks together with horizontal cracks, triggering coating delamination. The crack classification of individual coatings is presented in Table 1. A morphology of a typical crack denoted as type 3 is depicted in Figure 6.

### 3.4. Mechanical Properties

**Figure 7:**
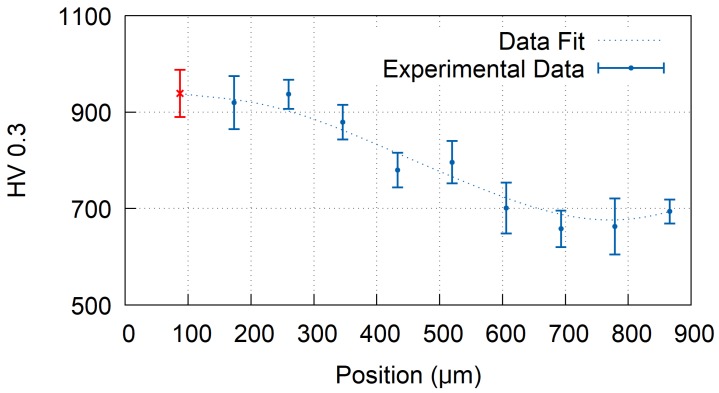
Microhardness profile of the laser heat treated sample L4. For samples with gradient hardness, the value closest to the surface (red color) was taken as reference.

To quantify the effect of thermal treatment, a microhardness of the coating was measured. The lowest microhardness of 639 ± 37 HV0.3 was measured for the as-sprayed sample, while the highest hardness was obtained for the furnace treated sample F1 with the average value 1156 ± 131 HV0.3. In case of surface treated samples, gradually changing values of microhardness were observed, with the highest hardness measured close to the coatings free surface and lowest hardness close to the substrate. An example of such microhardness profile is provided in Figure 7 for sample L4. The depth of the influenced layer varied significantly with the heat treatment parameters from a few tens of micrometers up to 800 μm (sample P2). To facilitate a mutual comparison of all coatings, the microhardness, measured closest to the coating free surface (corresponding to the depths of approximately 87 ± 5 μm below the surface, indicated by the red cross in Figure 7), was selected as a reference value. The average values of microhardness, calculated from five indents, are listed in Table 1.

**Figure 8:**
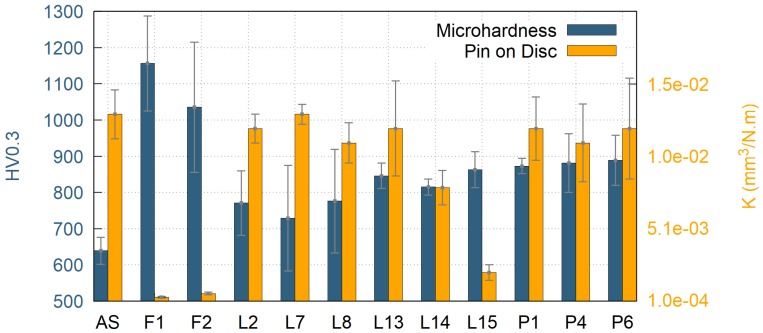
Microhardness and Pin on Disc volume loss for as-sprayed, furnace treated, laser treated and plasma treated samples.

Pin on Disc wear resistance was evaluated for selected samples. The criteria for samples selection were based on following parameters: (i) significant change in surface microhardness and (ii) cracks in the coating of type 0 or 1 only. Based on these criteria, nine surface heat treated samples were selected, along with the as-sprayed sample and two furnace heat treated samples as the reference. The determined values of wear resistances of the coatings, presented by material volume loss K, are listed in Table 1. Graphical interpretation of hardness and wear resistance of the samples is pictured in Figure 8.

## 4. Discussion

**Figure 9:**
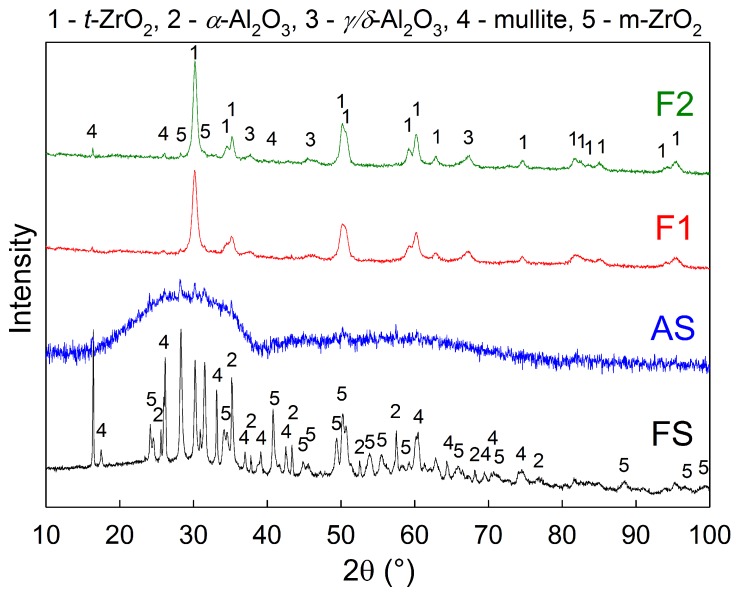
X-ray diffraction patterns of the feedstock (FS), as sprayed coating (AS), and furnace heat treated samples (F1 and F2).

The original crystalline feedstock powder was transformed during spraying to almost fully amorphous coatings, as can be seen from the diffraction patterns in Figure 9 and Table 2, where FS and AS stand for feedstock powder and as-sprayed coating, respectively. Subsequently, the amorphous phase was partially transformed back to crystalline during heat treatment, forming mainly t-ZrO_2_ nano-crystals, together with δ-Al_2_O_3_ and m-ZrO_2_. Silicon dioxide, present in the original feedstock, remained in the amorphous phase, or transformed to mullite (3Al_2_O_3_·2SiO_2_), depending on the heat treatment conditions of the samples.

The furnace heat treatment resulted in almost fully crystalline samples (87% and 100% crystallinity for samples F1 and F2, respectively). As expected, both of these samples showed the highest Vickers microhardness values 1156 ± 131 HV0.3 for sample F1 and 1035 ± 180 HV0.3 for sample F2, as well as the best wear resistant behaviour with material volume loss of 2.9 × 10^−4^ mm^3^/N·m for sample F1 and 5.4 × 10^−4^ mm^3^/N·m for sample F2 (see Table 1 for wear rate results of all samples). This was due to the fact that, during the crystallization, the coatings could freely undergo unconstrained shrinkage, since they were removed from the substrate prior the heat treatment. Consequently, there was no CTE mismatch between the substrate and the coating, resulting in no additional cracking. Furthermore, some micro-cracks originally present in the as-sprayed material closed up by the sintering effect during the heat treatment, which improved the mechanical properties as well. However, the highest contribution to the observed increase in microhardness and wear-rate resistance may be attributed to formation of nanocrystallites of various phases within the microstructure. In particular, a formation of t-ZrO_2_ is believed to have a significant influence on the improvement of mechanical properties. In order to conceive fine differences in the microstructure, crystallite size (or coherently diffracting domains’ size) of the t-ZrO_2_ phase was determined by the Rietveld refinement method for XRD diffractograms (see Table 2). Crystallite size of the t-ZrO_2_ phase of the sample F1 showed the smallest crystallite size (14 nm) from all the measured samples.

**Figure 10:**
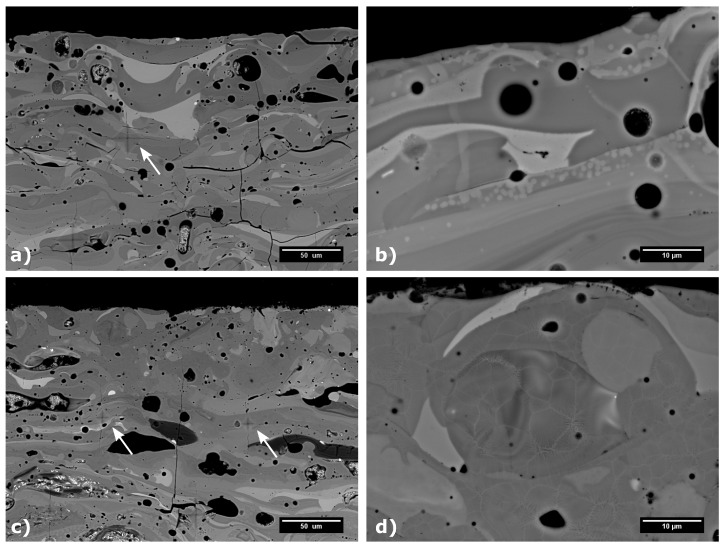
Comparison of the samples heat treated by laser—sample L15 (**a**,**b**) and plasma—sample P2 (**c**,**d**). Vickers indents marked by the arrows.

Laser heat treated samples showed significant formation of vertical cracks, originating from the constrained shrinkage during the crystallization. Therefore, samples with no cracks or short vertical cracks only were selected for further tests, since extensive cracking of the samples may compromise its overall mechanical properties, corrosion and chemical resistance. The best mechanical properties were measured for the samples L14 and L15, which exhibited rather low wear rates of 7.9 × 10^−3^ mm^3^/N·m and 2.0 × 10^−3^ mm^3^/N·m, respectively. For these samples, the highest transverse velocity of the laser of 800 mm/min was used, combined with the highest laser powers of 1100 W and 1300 W, respectively. These two samples showed only limited cracking, significant increase in Vickers microhardness up to 863 ± 50 HV0.3 for sample L15 and improvement in wear resistant properties (compared to the as-sprayed coating). In fact, the sample L15 showed the best wear resistance from all surface heat treated samples. Similarly to the furnace treated samples, the sample L15 transformed to fully crystalline material, in the vicinity of the coatings surface, with the average CDD of t-ZrO_2_ of 32 nm. The change in the microstructure and the appearance of Vickers indents are presented in Figure 10a,b. From Figure 10, formation of bright domains within individual splats was observed in sample L15. These may be segregated domains of ZrO_2_ phase; however, they are too small for an accurate identification by EDX. Interestingly, the sample L14 remained mostly amorphous, with only 17% of the sample surface crystallized, as evaluated by the Rietveld analysis of XRD patterns measured from the sample surface. The rather incomplete crystallization was caused by the fact that the combination of 800 mm/min transverse velocity and lower power of 1100 W heated up the sample’s surface just a little above the crystallization temperature. Due to cooling through substrate heat transfer, the crystallization was very limited in this case, which in turn leads to inferior wear resistance, in comparison with the sample L15.

**Figure 11:**
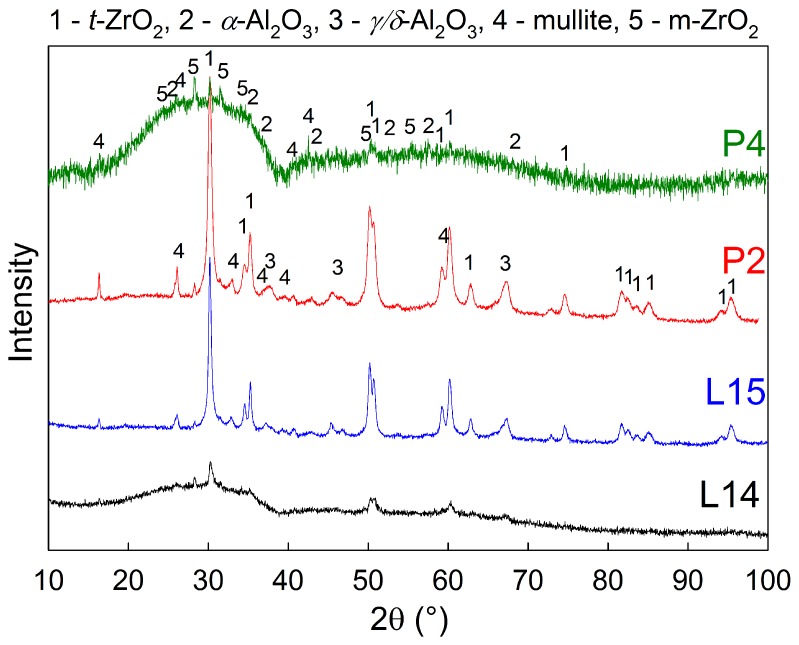
X-ray diffraction patterns of the laser and plasma heat treated samples.

Plasma heat treatment resulted in an increase of the Vickers microhardness for the samples P1, P2, P4 and P6. Surprisingly, the related improvement of wear resistance of plasma heat treated samples was not so pronounced, compared to the as-sprayed sample. This was probably caused by the used high transverse velocity selected to prevent melting of the samples’ surfaces. The high plasma torch movement speeds resulted in very short time of treatment, during which the samples were exposed to the temperatures above the crystallization point. Consequently, the grain nucleation and diffusion growth processes were limited and possibly happened only on the very specimen surface. The XRD patterns of all but one of the plasma treated samples showed only minor changes, compared to the as-sprayed samples. The only difference was the sample P2, which was produced using the highest plasma power of 150 kW and lowest transverse velocity of 3000 mm/min and its XRD pattern suggest 100% surface crystallinity, comparable to the laser treated sample L15 (see the Figure 11). However, the wear resistance properties were not measured for the P2 sample, since the most excessive cracking was observed in the SEM (see the Figure 6). In between the cracks, the cross-section of the sample P2 showed a microstructure (Figure 10) similar to the one of furnace treated samples F1 and F2. Formation of dark domains of alumina, enclosed by lighter regions, rich in ZrO_2_ were observed in back scattered electron mode in SEM and such element distribution was confirmed by local EDX analysis. The remaining plasma heat treated samples didn’t show any changes in the phase composition (compared to the as-sprayed state) and, therefore, no microstructure changes were observed in SEM for them.

Plasma surface heat treatment of the ceramic coating is very challenging. The power density of high enthalpy plasma torch, combined with low thermal conductivity of the ceramic coatings needs precise adjustment of heat treating conditions to provide sufficient heat treatment of the coating and, at the same time, prevent the coating from undesirable overheating (or even remelting). Therefore, further optimization of plasma heat treatment conditions, e.g., a change in the stand-off distance, or the use of multiple short passes of the plasma torch above the coating, has the potential to result in similar improvement of mechanical properties, such as was presented for the samples heat treated by laser. The analysis of the wear tracks in the SEM showed remarkable differences in the wear mechanism. The as-sprayed sample displayed rather wide (over 3 mm) wear track, reflecting its high material removal rate in the Pin on Disc test. The observed wear mechanism was mainly debonding and cracking of loosely connected splats which were crushed by the sliding ball, leaving coarse debris in the wear track. On the other hand, the furnace-treated samples showed shallow and narrow wear track (about 1.1 mm in width) filled with fine debris originating mainly from grinding off of the sintered splats. No splat debonding was observed for the furnace-treated sample. A combination of both above-mentioned mechanisms was observed for laser and plasma treated samples, where the wear tracks were filled with mixture of fine and coarse wear debris, the former originating from grinding off of the surface, and the latter formed due to debonding and cracking of splats.

## 5. Conclusions

Ceramic powder of near eutectic composition from the ternary system of Al_2_O_3_−ZrO_2_−SiO_2_ was plasma sprayed onto steel substrates to create 1 mm-thick coatings. Atmospheric plasma spraying was carried out by hybrid water stabilized plasma torch WSP-H 500. The as-sprayed coatings were amorphous and their hardness and wear resistance are rather low. Unfortunately, bulk furnace treatment is not applicable for coatings on metallic substrates, since it irreversibly deteriorates the properties of the substrate and causes cracking and spallation of the coating due to CTE mismatch and substrate oxidation. Therefore, the coatings were subjected to surface heat-treatment to improve mechanical properties of the coating while maintaining good adhesion to the substrate. In addition, the stripped-off coatings were subject to furnace heat treatment to obtain reference samples. The surface heat treatment by laser or plasma torch resulted in significant changes in the coating microstructure. The surface layer of the coating transformed from amorphous to nanocrystalline structure within the splats. Some of the heat treatment conditions led to formation of vertical cracks in the coatings, which compromised their overall mechanical properties. In other cases, these changes were accompanied by improvement of the coating mechanical properties. Vickers microhardness increased from 639 ± 37 HV0.3 for the as-sprayed coating, up to 1156 ± 131 and 1129 ± 169 HV0.3 for the furnace treated and selected plasma treated samples, respectively. Wear resistance was improved more than six times, from the value of material volume loss 1.3 × 10^−2^ mm^3^/N·m of the as-sprayed sample, down to 2.0 × 10^−3^ mm^3^/N·m for some laser treated samples. The changes in mechanical properties of the heat treated samples were caused by the solid stage crystallization in the surface layer of the originally amorphous coatings. The samples with the highest hardness and wear resistance were fully crystalline, and had a very low size of coherently diffracting domains in the range of 14–32 nm. Utilization of surface heat treatment could be an efficient final stage of coating manufacturing, since only a thin surface layer of the coating can be treated to meet the specifications for targeted wear-resistant application. Heat treatment of Al_2_O_3_−ZrO_2_−SiO_2_ coatings is a very stochastic process as each splat has different chemical composition. Therefore, solid state crystallization has different kinetics among splats, giving rise to various phases and crystalline grain sizes. Improvement of mechanical properties is then controlled mainly by formation of crystallites with sizes in tens of nanometers. Surface treatment of ceramic coatings by laser and plasma, presented in this study, was successfully used for inducing such nanocrystalline microstructure in originally amorphous material and these two methods may find application for similar materials, which tend to form amorphous coatings.

## Figures and Tables

**Table 1:** Used parameters of heat treatment and corresponding microhardness and wear resistance esults.

	**Dwell Time (min)**	**Temperature (°C)**	**HV0.3**	**Cracks ^1^**	**K (mm^3^/N·m)**
AS	–	–	639 ± 37	0	1.3 e-2
F1	1	1050	1156± 131	0	2.9 e-4
F2	5	1050	1035 ± 180	0	5.4 e-4
	**Transverse velocity (mm/min)**	**Power (W)**	**Surface HV0.3**	**Cracks**	**K (mm^3^/N·m)**
L1	50	250	632 ± 45	0	–
L2	50	300	771 ± 89	1	1.2 e-2
L3	50	350	906 ± 87	2	
L4	50	400	939 ± 85	2	
L5	200	250	801 ± 86	0	
L6	200	300	715 ± 107	0	
L7	200	350	729 ± 146	0	1.3 e-2
L8	200	400	776 ± 143	1	1.1 e-2
L9	200	450	819 ± 143	2	–
L10	200	500	869 ± 35	3	–
L11	800	500	671 ± 146	0	–
L12	800	600	688 ± 12	0	
L13	800	800	846 ± 35	0	1.2 e-2
L14	800	1100	815 ± 22	1	7.9 e-3
L15	800	1300	863 ± 50	1	2.0 e-3
P1	3000	100,000	873 ± 21	0	1.2 e-2
P2	3000	150,000	1129 ± 169	3	–
P3	6000	100,000	645 ± 132	0	–
P4	6000	150,000	881 ± 81	1	1.1 e-2
P5	12,000	100,000	667 ± 47	0	–
P6	12,000	150,000	889 ± 69	0	1.2 e-2

0—no cracks, 1—short vertical cracks, 2—long vertical cracks, 3—vertical and horizontal cracks.

**Table 2:** Crystallinity of the samples. Ratio of t-ZrO_2_ and its CDD size.

	Amorphous	Crystalline	t-ZrO_2_	
	(%)	(%)	(%)	CDD (nm)
FS	16	84	10	40
AS	92	8	5	–
F1	13	87	33	14
F2	0	100	35	16
L14	83	17	29	21
L15	0	100	46	32
P2	0	100	41	23
P4	92	8	5	–
